# Influence of Etching Trench on $K_{eff}^{2}$ of Film Bulk Acoustic Resonator

**DOI:** 10.3390/mi13010102

**Published:** 2022-01-08

**Authors:** Chao Gao, Yang Zou, Jie Zhou, Yan Liu, Wenjuan Liu, Yao Cai, Chengliang Sun

**Affiliations:** 1 The Institute of Technological Sciences, Wuhan University, Wuhan 430072, China; GaoChao96@whu.edu.cn (C.G.); yang_zou@whu.edu.cn (Y.Z.); zhoujie_ist@whu.edu.cn (J.Z.); liuyan92@whu.edu.cn (Y.L.); lwjwhu@whu.edu.cn (W.L.); 2 Hubei Yangtze Memory Laboratories, Wuhan 430072, China

**Keywords:** radio frequency (RF), MEMS, FBAR, effective electromechanical coupling coefficient

## Abstract

As radio-frequency (RF) communication becomes more ubiquitous globally, film bulk acoustic resonators (FBAR) have attracted great attention for their superior performance. One of the key parameters of an FBAR, the effective electromechanical coupling coefficient ($K_{eff}^{2}$), has a great influence on the bandwidth of RF filters. In this work, we propose a feasible method to tune the $K_{eff}^{2}$ of the FBAR by etching the piezoelectric material to form a trench around the active area of the FBAR. The influence of the position of the etching trench on the $K_{eff}^{2}$ of the FBAR was investigated by 3D finite element modeling and experimental fabricating. Meanwhile, a theoretical electrical model was presented to test and verify the simulated and measured results. The $K_{eff}^{2}$ of the FBAR tended to be reduced when the distance between the edge of the top electrode and the edge of the trench was increased, but the Q value of the FBAR was not degraded. This work provides a new possibility for tuning the $K_{eff}^{2}$ of resonators to meet the requirements of different filter bandwidths.

## 1. Introduction

The rapid development of wireless mobile communication has led to higher requirements for radio-frequency (RF) devices. As an important element in the RF front-end, filters ideally possess large bandwidths, have low insertion losses and can undergo miniaturization [1]. Film bulk acoustic filters have been attracting researchers’ attention in recent years, since the achievable high acoustic velocity, good temperature stability and large electromechanical coupling coefficients ($K^{2}$) of piezoelectric film materials, for example, aluminum nitride (AlN) and scandium-doped AlN (ScAlN), render them suitable to meet the harsh requirements of 5G wireless communication [2].

The effective electromechanical coupling coefficient ($K_{eff}^{2}$) of a film bulk acoustic resonator (FBAR) is a core parameter which influences the bandwidth and cutoff frequency of filters. An adequate $K_{eff}^{2}$ bringing in a large bandwidth is achievable by using suitable piezoelectric materials or doping methods which are commonly used in previous process technologies. J.-S. Moulet et al. reported BAW devices fabricated with thin single crystalline LiNbO_3_ films and realized a $K_{eff}^{2}$ greater than 30% [3]. M. Pijolat et al. demonstrated that the $K_{eff}^{2}$ of LiNbO_3_-based FBAR can be as high as 43% [4,5]. Milena Moreira et al. illustrated that the $K_{eff}^{2}$ of ScAlN-based FBAR showed a linear two-fold increase when the Sc concentration was increased from 0 to 0.15 [6]. It is reported that the piezoelectric coefficient can be increased by about five times when the proportion of scandium doped in aluminum nitride is 40% [7,8,9,10,11]. However, manipulating the cutoff points to better satisfy the demand of the frequency band is challenging, and needs the precise control of the $K_{eff}^{2}$ of the FBAR. Researchers have indicated that changing the thickness ratio of the electrode and piezoelectric material can affect the $K_{eff}^{2}$ of an FBAR. K.M. Lakin et al. reported that the $K_{eff}^{2}$ of an FBAR was equal to the $K^{2}$ of the piezoelectric material when the electrode thickness was zero, and reached a maximum of 6.5% when the thickness ratio of the electrode to piezoelectric material was about 0.1 [12]. Hao Zhang et al. demonstrated that the $K_{eff}^{2}$ was reduced from 6.9% to 5.0% when the thickness of Mo(top)/AlN/Mo(bottom) was changed from 0.28 μm/1.18 μm/0.28 μm to 0.46 μm/0.62 μm/0.46 μm, since the conversion efficiency between electrical energy and sound energy was reduced [13]. However, precise thickness control is difficult in actual processing. Adding external passive devices such as capacitors and inductors into circuits can also influence the $K_{eff}^{2}$ of an FBAR. Qingrui Yang et al. indicated that the 3 dB bandwidth of an AlN-based bulk acoustic wave (BAW) filter can be up to 12.3% under the influence of adding auxiliary inductors to the lattice topology, which was more than twice that of the conventional AlN-based BAW filter [14]. Paras Chawla et al. indicated that the introduction of inductors in the filter topology increased the bandwidth of a BAW filter, while the introduction of capacitors did the opposite and reduced the bandwidth of the filter [15]. However, the introduction of passive devices has a great impact on the Q value of the resonator and causes an increase in the filter volume.

In this paper, the piezoelectric material was etched to form a trench around the active area of resonators and the trench can be equivalent to an external capacitor. The value of the external capacitor was adjusted by changing the position of the etching trench. The influence of the etching trench on the $K_{eff}^{2}$ of the FBAR was investigated by using the finite element method (FEM) and FBAR devices with different trench positions were also manufactured for further verification. Both the simulated and measured results indicated that the $K_{eff}^{2}$ of the FBAR decreased as the distance (*d*) between the trench and the edge of the electrode increased; the measured $K_{eff}^{2}$ was 18.3% when *d* was 0 but dropped to 14.6% when *d* was 12 μm. Additionally, the Q value of the FBAR was calculated and normalized; the results show that it nearly remained unchanged. We have proved that the etching trench can indeed tune the $K_{eff}^{2}$ of the FBAR without degenerating the Q value of the FBAR. The $K_{eff}^{2}$ of the FBAR can be well adjusted with the etching trench in the fabricating process, which can be potentially used in future work.

## 2. Materials and Methods

**Figure 1:**
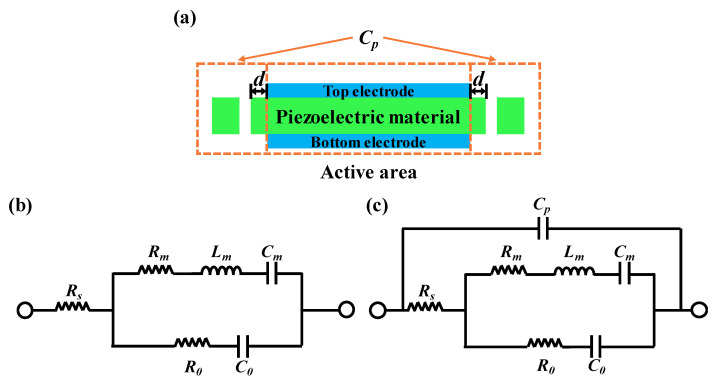
(**a**) Cross-sectional structure of FBAR with the etching trench. (**b**) The MBVD model of FBAR without $C_{p}$. (**c**) The MBVD model with $C_{p}$.

Figure 1a shows the cross-sectional view of the FBAR with the etched trench; the region where the top and bottom electrodes overlap with the piezoelectric material is the active area of the FBAR. The introduction of the trench can be equivalent to a parallel capacitor ($C_{p}$) connecting with the resonator.

For FBAR devices, the $K_{eff}^{2}$ is calculated as [16]:

(1) $$K_{eff}^{2}=\frac{\pi^{2}}{4}\frac{f_{s}}{f_{p}}\frac{f_{p-}f_{s}}{f_{p}},$$

where $f_{s}$ and $f_{p}$ are the series and parallel resonant frequencies, respectively. The MBVD (Modified Butterworth–Van Dyke) model [17], as shown in Figure 1b, can be used to express the electric characteristics of the FBAR. The model consists of six elements: $C_{0}$ is the static capacitance, $C_{m}$ is the motional capacitance, and $L_{m}$ is the motional inductance. The remaining parameters $R_{m}$, $R_{s}$ and $R_{0}$ represent the motional resistance, electrode ohmic loss parasitic and acoustic loss parasitic, respectively. The series $R_{m}$-$C_{m}$-$L_{m}$ circuit branch reflects the interaction between the RF (radio-frequency) signal and the bulk acoustic wave. The series frequency of the FBAR can be expressed in terms of $L_{m}$ and $C_{m}$, and the shunt frequency of the FBAR can be expressed in terms of $L_{m}$, $C_{m}$ and $R_{m}$. The calculations of $f_{s}$ and $f_{p}$ are as follows [18]:

(2) $$f_{s}=\frac{1}{2\pi \sqrt{L_{m}C_{m}}},$$

(3) $$f_{p}=\frac{1}{2\pi }\sqrt{\frac{C_{m}+C_{0}}{C_{m}C_{0}L_{m}}}=f_{s}\sqrt{\frac{C_{m}}{C_{0}}+1},$$

As shown in Figure 1c, we proposed an MBVD model with the external parallel capacitor to describe the electric characteristic of the FBAR with the etching trench shown in Figure 1a. Using the MBVD model, we found that $f_{s}$ remains unchanged and $f_{p}$ can be expressed as:

(4) $$f_{p}=\frac{1}{2\pi }\sqrt{\frac{C_{m}+C_{0}+C_{p}}{C_{m}L_{m}\left(C_{0}+C_{p}\right)}}=f_{s}\sqrt{\frac{C_{m}}{C_{0}+C_{p}}+1},$$

According to Formulas (1), (2) and (4), the $K_{eff}^{2}$ of the FBAR can be tuned by changing $C_{p}$.

**Figure 2:**
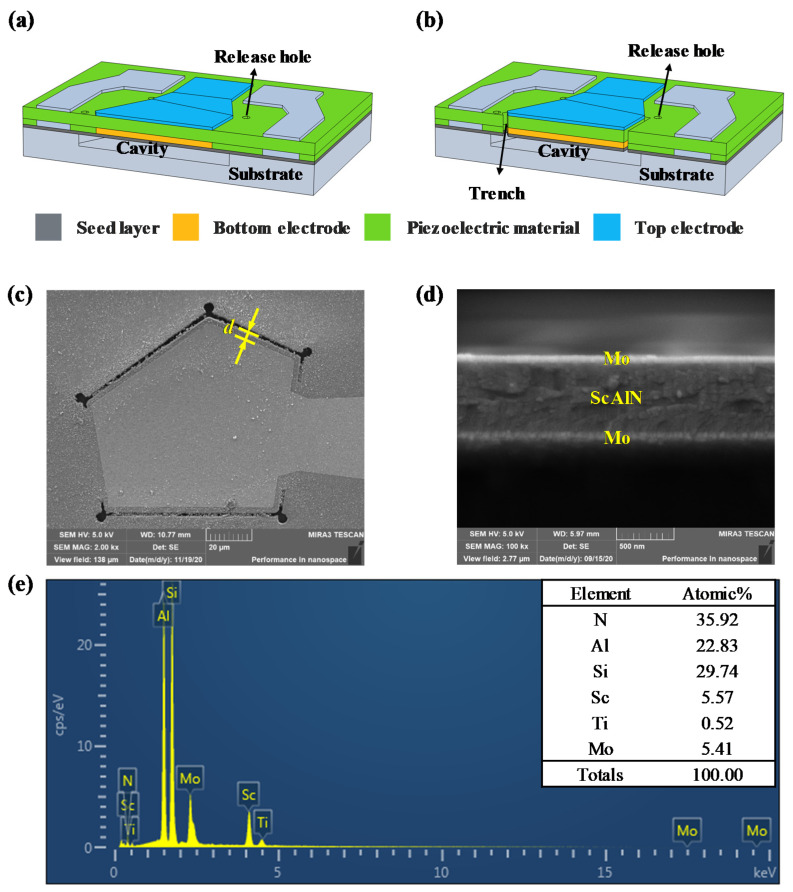
3D cross-sectional schematics: (**a**) Traditional FBAR and (**b**) FBAR with trench. Scanning electron microscope (SEM) images for the fabricated FBAR: (**c**) Top view of FBAR with trench. (**d**) Cross-sectional view of FBAR with trench. (**e**) Energy-dispersive spectroscopy (EDS) elemental spectrum and quantification results.

A 3D finite element model was built in finite element simulation software COMSOL Multiphysics to simulate the influence of the etching trench on the performance of the FBAR. Figure 2a,b show the 3D cross-sectional schematics of the traditional FBAR and the FBAR with the etching trench, respectively. The difference between these two types of resonators is that the piezoelectric material of the latter is etched to form a trench for changing the $K_{eff}^{2}$ of the resonator. The distance *d* between the edge of the trench and the edge of the top electrode was set to values of 0 μm, 2 μm, 4 μm, 8 μm and 12 μm, to change the value of $C_{p}$, but the width of the trench remained constant at 2 μm. The FBAR structure of the 3D model was a layer stack of seed Ti layer, bottom Mo electrode, middle Sc_0.20_Al_0.80_N piezoelectric layer and top Mo electrode. The thicknesses of the piezoelectric film, the bottom/top electrode and the seed layer were 580 nm, 50 nm and 20 nm, respectively.

We also fabricated the FBAR devices to test and verify the simulated results. Each layer of the resonator was processed to the same thickness as the 3D simulation model, and the distance *d* was processed to a variable from 0 to 12 μm, while the width of the trench was processed to the same fixed value of 2 μm as per the 3D simulation model defined. Figure 2c,d show the top and cross-sectional SEM images of the resonator with the etching trench, respectively. Figure 2e shows the energy-dispersive spectroscopy (EDS) elemental results of the devices; the atomic percent indicates that the scandium-doping ratio was 20%.

**Figure 3:**
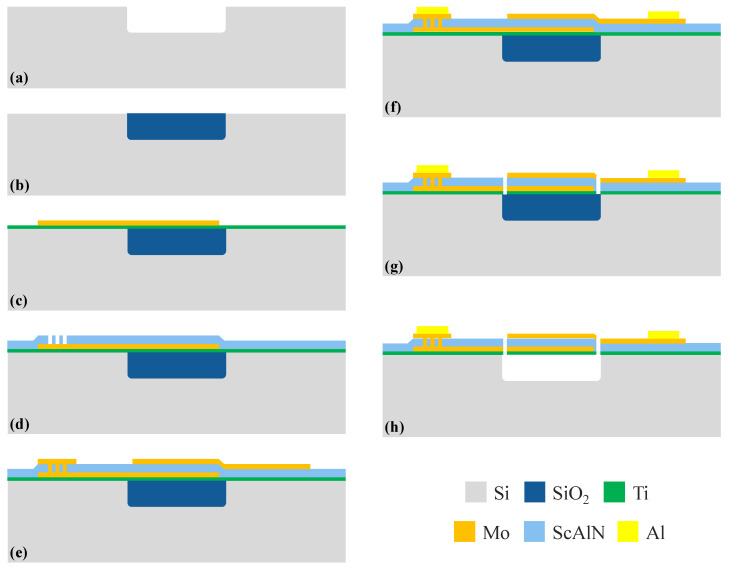
ScAlN FBAR fabrication process flow diagram: (**a**) Etching swimming pool on high-resistance silicon. (**b**) Deposit SiO_2_ and CMP. (**c**) Deposit Ti and Mo, pattern. (**d**) Deposit ScAlN, pattern. (**e**) Deposit Mo, pattern. (**f**) Deposit Al pad, pattern. (**g**) Etch release holes and trench. (**h**) VHF release SiO_2_.

The detailed fabrication process is illustrated in Figure 3. The fabrication process started with etching a silicon substrate to form a swimming pool which functioned as an air reflection interface of bulk acoustic waves. Then, silicon dioxide (SiO_2_) was deposited on the substrate as a sacrificial layer to fill the entire air cavity by low-pressure chemical vapor deposition (LPCVD). Chemical–mechanical polishing (CMP) stopping on silicon was used to remove superfluous SiO_2_. Next, a 20 nm-thick seed Ti layer was deposited to obtain a piezoelectric film with better quality, and a 50 nm-thick bottom Mo electrode was deposited and patterned. Then, 580 nm-thick Sc_0.20_Al_0.80_N was deposited by magnetron sputtering, and the ScAlN thin film was etched by inductively coupled plasma (ICP) dry etch with an etching rate of 90 nm/min, so that connecting holes were formed. After that, a 50 nm-thick top Mo electrode was deposited and patterned. Subsequently, a 1 μm-thick Al electrode was deposited and patterned to serve as the electrode pad. Finally, release holes and trench were formed by etching the ScAlN again, and VHF (vapor hydrofluoric acid) dry etching was used to etch the SiO_2_ in the swimming pool, thus releasing the whole FBAR device.

## 3. Results and Discussion

**Figure 4:**
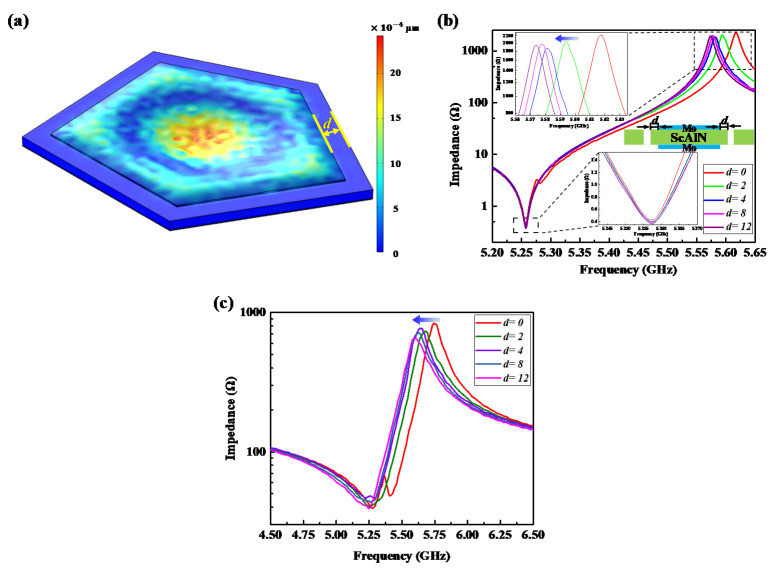
(**a**) The displacement shape at resonant frequency. Impedance curves of FBARs with the distance *d* changed: (**b**) Simulated results. (**c**) Measured results.

The displacement shape at resonant frequency is shown in Figure 4a; it is the thickness vibration mode. The simulated frequency response of the FBAR at different distances *d* is presented in Figure 4b; the distance *d* was set to 0, 2 μm, 4 μm, 8 μm or 12 μm. As shown in Figure 4b, the series resonant frequency of the FBAR was 5.257 GHz and the shunt resonant frequency was at 5.617 GHz when *d* was 0. As the distance *d* increased, all the series resonant frequencies of the FBARs were still at 5.527 GHz, but the parallel resonant frequencies became smaller. The measured frequency responses presented in Figure 4c also prove the same influence of distance *d* on the position of the resonant frequency.

**Figure 5:**
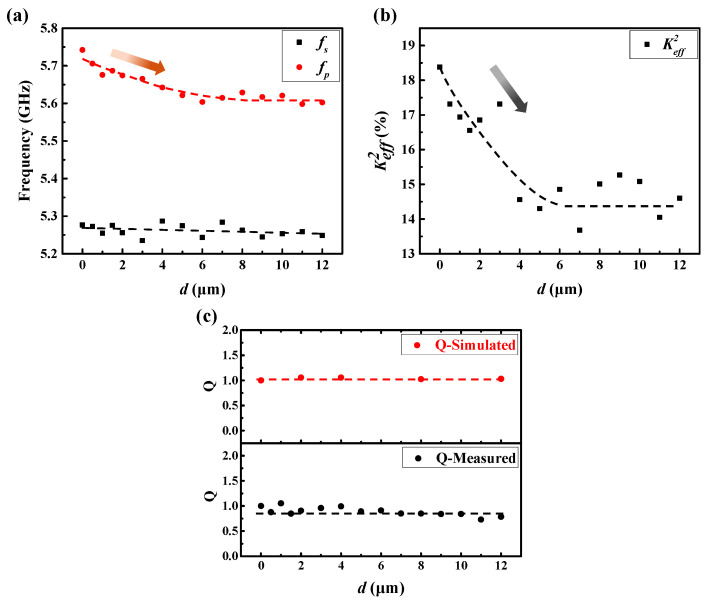
(**a**) The detailed frequency change of measured results with different distance d. (**b**) The calculated $K_{eff}^{2}$. (**c**) The normalized Q values of FBARs with different distances *d*.

The detailed frequency change of the measured results can be seen in Figure 5a. $f_{s}$ remained almost unchanged, and $f_{p}$ dropped slowly first and tended to become a constant with the distance *d* increased. Figure 5b shows the tendency of the calculated $K_{eff}^{2}$ by Formula (1); $K_{eff}^{2}$ was 18.3% when *d* was 0 and decreased to 14.6% when *d* was 12 μm. Compared with the electrical loss, the loss caused by the etching trench can be very small; therefore, the influence of the etching trench on the Q factor will be relatively small. Figure 5c demonstrates the variation rule of the normalized Bode-Q [19] value of the simulated and measured results. Both the simulated and measured results note the same trend that the Q value remained unchanged when *d* was increased, which means the influence of the etching trench on the Q value of the FBAR can be ignored.

**Figure 6:**
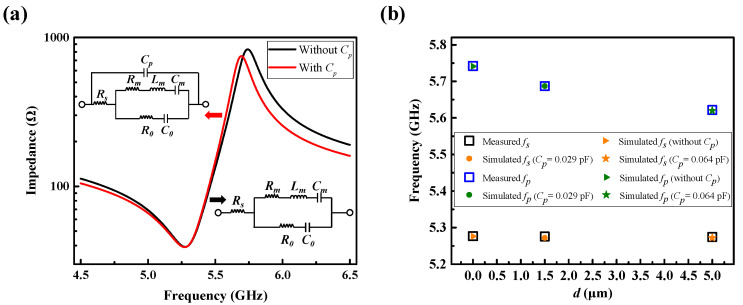
(**a**) Comparison of impedance curves with and without an external capacitor connected in parallel with a resonator. (**b**) Measured frequencies of resonators with different distance (*d*) are fitted by capacitors ($C_{p}$).

Figure 6a shows that $f_{p}$ was reduced but $f_{s}$ remained unchanged when an external capacitor was in parallel with the resonator. As shown in Figure 6b, the measured resonance frequencies with *d* equal to 0, 1.5 μm or 5 μm, can be well fitted by the MBVD model with different external $C_{p}$. Generally, the variation of distance *d* can be regarded as equivalent to the alteration of external $C_{p}$.

## 4. Conclusions

In this work, we proposed using etching piezoelectric material to form a trench near the active area of an FBAR to tune $K_{eff}^{2}$. The influence of the etching trench on the FBAR was investigated using FEM and fabricating resonators with the etching trench located at different positions. The parallel resonant frequency and $K_{eff}^{2}$ of the resonator were influenced by the etching trench position and decreased as the distance *d* increased, but the effects of the etching trench position on the series resonant frequency and the Q value were negligible. We also theoretically proposed a modified electrical model to verify the simulated and measured results; the introduction of the etching trench can be equivalent to an external $C_{p}$ connection with the resonator. Using Sc_0.20_Al_0.80_N as the piezoelectric functional material, an FBAR with adjustable $K_{eff}^{2}$ of 14% to 18.3%, controlled by the position of the etching trench, was obtained. The proposed etching trench in piezoelectric materials can be compatibly achieved in the fabrication process and provides a potential solution for finely adjusting the bandwidth of filters in the future.

## Data Availability

Data is contained within the article.
